# Investigating fouling at the pore-scale using a microfluidic membrane mimic filtration system

**DOI:** 10.1038/s41598-019-47096-6

**Published:** 2019-07-22

**Authors:** Nandini Debnath, Aloke Kumar, Thomas Thundat, Mohtada Sadrzadeh

**Affiliations:** 1 Department of Mechanical Engineering, 10-367 Donadeo Innovation Centre for Engineering, Advanced Water Research Lab (AWRL), University of Alberta, Edmonton, T6G 1H9 Canada; 2 Department of Mechanical Engineering, Indian Institute of Science, Bangalore, India; 3 Department of Chemical and Materials Engineering, University of Alberta, Edmonton, T6G 2G8 Canada; 4 Department of Chemical and Biological Engineering, School of Engineering and Applied Sciences, University of Buffalo, Buffalo, 14260 USA

**Keywords:** Fluidics, Chemical engineering, Mechanical engineering

## Abstract

The work investigates fouling in a microfluidic membrane mimic (MMM) filtration system for foulants such as polystyrene particles and large polymeric molecules. Our MMM device consists of a staggered arrangement of pillars which enables real-time visualization and analysis of pore-scale phenomena. Different fouling scenarios are investigated by conducting constant-pressure experiments. Fouling experiments are performed with three different types of foulants: polystyrene particle solution (colloidal fouling), polyacrylamide polymer solution (organic fouling) and a mixture of these two solutions (combined fouling). Four major categories of microscopic fouling are observed: cake filtration (upstream), pore blocking (inside the pores), colloidal aggregation (downstream) and colloidal streamer fouling (downstream). Our microfluidic experiments show that downstream colloidal aggregation and streamer fouling have a significant effect on overall membrane fouling which were not studied before.

## Introduction

Membrane filtration processes, such as microfiltration (MF) and ultrafiltration (UF), have been widely used across a broad range of industries including wastewater treatment^1^, effluent treatment^2^, removal of pharmaceuticals^3^, food processing^4^, and production of reusable and potable water^5–7^. Fouling of membranes represents a singular issue and limiting condition in the deployment of membrane-based filtration systems^8^. Fouling generally occurs by the attachment of the water constituents on the surface or within the pores of the membrane, resulting in dramatic reduction in flux over time^9^. The fouling propensity depends on the hydrodynamics (flux, pressure and flow velocity), feed solution properties (foulant types, concentration, pH, and ionic strength), surface interactions (surface charge and polarity) and membrane morphology (pore size and shape)^10,11^.

Membrane fouling has been intensely studied^9,12,13^ and various mechanisms of membrane fouling have been identified. Amongst the various mechanisms, cake layer/gel formation^14–16^ and pore blocking^17,18^ are usually regarded as the major fouling mechanisms; cake-layer formation occurs at the upstream end of the membrane, while pore blocking occurs at the pore-scale of the membrane^19–21^. A common method to investigate the fouling mechanism is to evaluate the flux decline over filtration time. The various mechanisms of membrane fouling exhibit a signature decline of flux with time^22,23^. *Ex-situ* fouling tests provide valuable insight into the effect of different parameters on fouling; however, the evolution of fouling on the surface and within the pores cannot be studied.

Recently, a large number of studies has been devoted to elucidating the transport of particle-laden flow in a porous media^11,12,24–26^. In this regard, microfluidic mimics of membranes have become an important experimental platform for investigating fouling at the pore-scale^11,24,25^. Specifically, photolithography can be easily adapted to designing microfluidic membrane mimic (MMM) systems with a pore length-scale comparable to the pore size of MF membranes^11,27,28^. MMM systems have a significant advantage in that they allow for an easy integration with various sensing platforms, such as optical microscopy. This allows for an *in-situ* and real-time visualization of various fouling processes operative at the pore-scale^28^. This alleviates the challenge faced with a large-scale membrane filtration system, where typically only end-point visualization is possible. Thus, MMM system enables investigating the effect of hydrodynamic conditions (initial flux and pressure) on fouling and analyzing the physiochemical interactions responsible for fouling phenomena at pore scale^29,30^. Debnath and Sadrzadeh^11^ reviewed the use of MMM devices in the context of membrane fouling. The use of MMM devices has shed light on downstream fouling, which refers to the fouling when foulants can pass through the skin layer of denser structure and accumulate at the downstream stagnation corners of the skin layer pores^11^. Sendekie *et al*. observed accumulation of the polystyrene particles (0.5 µm) at the downstream corners of the micro-pillars^31^. Bacchin *et al*. interpreted this downstream deposition with cluster growth kinetics, where the constructive and destructive cluster-cluster interactions play an important role on the aggregation process in a microchannel^32^. Despite these advantages, the use of MMM devices to understand fouling at the pore-scale has been limited and specifically the issue of downstream fouling needs to be investigated in greater detail.

In this work, a systematic fouling study was conducted using three types of foulants colloidal particles, polymer and a mixture of these two to investigate combined fouling in an MMM system. Our MMM device consists of a staggered arrangement of micro-pillars mimicking an MF membrane pore size. The pore length-scale employed was 2 μm, which is one of the smallest gaps that can be reliably fabricated using the photolithography process. We examine the interplay between hydrodynamics and physiochemical interactions with a direct visualization of the MF process using optical microscopy. Constant-pressure experiments are performed in the dead-end mode using a Microfluidic Flow Control System (MFCS).

## Results and Discussion

### Microscopic membrane fouling

**Figure 1:**
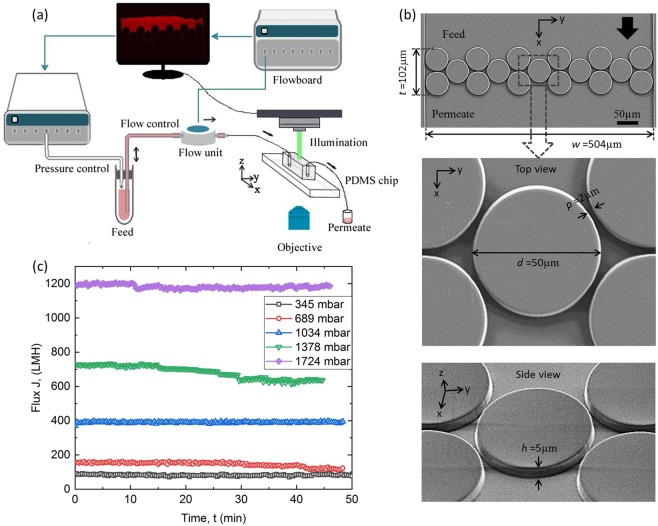
(**a**) A schematic of the experimental setup. A pressure controller and flow-board unit controls the pressure and the flow rate of the membrane mimic microfluidic device, respectively. The pressure difference causes the feed to enter one inlet of the device via the flow unit and the waste is collected from the outlet/permeates side. **(b)** SEM image of the microfluidic membrane mimic device with dimensions (membrane thickness, *t* = 102 µm and membrane width *w* = 504 µm). The blown-out sections show a gap, *p* = 2 µm, between any two pillars with diameter *d* = 50 µm (top view mode) and a height of *h* = 5 µm (side view mode). **(c)** Control experiments with ultrapure water shows almost constant flux at various pressures for the microfluidic membrane mimic device.

Constant-pressure and constant-flow rate filtration experiments were conducted using the MMM device shown in Fig. 1(a). All experiments were performed at room temperature and neutral pH. As discussed earlier, our MMM device consists of an array of pillars with height, *h* = 5 µm and pillar gap, *p* = 2 µm, which provides hydraulic diameter of 2.8 µm and provides a pore diameter comparable with the pore size of typical MF membranes (0.1–10 µm) (Fig. 1(b)). Before using the microchips as an MF membrane mimic, control experiments were performed at constant pressure using Millipore water to ensure the integrity of the microfluidic device. Figure 1(c) shows that, in the absence of fouling, the water flux was almost constant at different pressures over time. In an MF process, pure water flux varies linearly with pressure. However, from our experimental data, water flux increased non-linearly with increasing pressure (Fig. S1 in Supporting Information). Given the pore size of our MMM device (∼2 µm), we might have exceeded the pressure threshold for filtration. As can be observed in Fig. S1, our MMM device showed more non-linear behavior at pressures greater than 689 mbar (10 psi). In addition, our MMM device may not be perfectly sealed at higher pressures. Hence, a constant pure water flux test is performed before starting any experiment. Constant pressure water flux results were repeated and the plot is shown in the supplementary (see Supplementary Fig. S2). It must be noted that control experiments were conducted before all filtration experiments and constant water flux was achieved each time prior to running the device with water containing foulant materials. Three types of synthetic wastewater solutions were prepared: polymer solution (PAM 0.2% w/w), particle solution (PS 0.2% w/w) and a combined solution (PAM + PS (0.2%) = 1:4 v/v). Polymer solution (PAM 0.2% w/w) was prepared by dissolving 1 g of anionic polyacrylamide (PAM: A-8354, 22 MDa, Kemira, AB, Canada) into 500 mL of DI water. Details about the foulants material preparation are provided in the materials and methods section. In constant-pressure experiments the feed solution is forced through the pillar-array and the foulant accumulates around the pillars over time. A total of 120 mins of filtration time was considered, unless otherwise stated. This fouling phenomenon caused a decrease in permeate flux for constant-pressure experiments with the three foulants.

**Figure 2:**
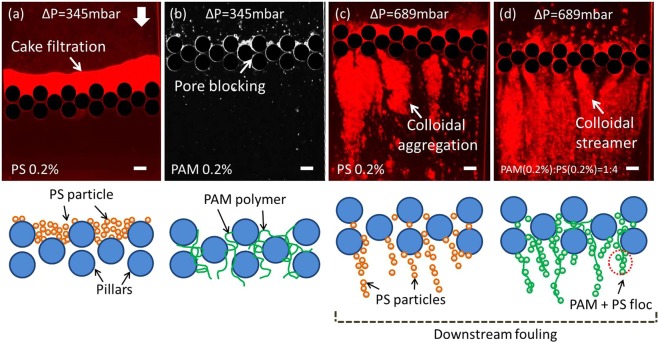
Microscopic colloidal fouling phenomena at different locations with their corresponding schematics. **(a)** cake filtration for PS 0.2% at 345 mbar at upstream, **(b)** pore blocking for PAM 0.2% at 345 mbar at the pillar pores, **(c)** colloidal aggregation for PS 0.2% at 689 mbar and **(d)** colloidal streamer formation for the combined fouling (PAM (0.2%):PS(0.2%) = 1:4) at 689 mbar at downstream. All images are taken at 120 min of filtration and the scale bars are 50 µm. Schematics are not to scale.

Figure 2 summarizes the observed fouling under different constant-pressure experiments. At low flow rates or pressures (Fig. 2(a,b)), the foulants started depositing on the surface or within the pores of the pillars. Cake filtration was generally observed for colloidal suspension (PS 0.2% w/w, amine-coated PS bead with 0.2 µm diameter) and pore blocking was observed for the polymer solutions (PAM 0.2% w/w). However, in the case of higher flow rates or pressures (Fig. 2(c,d)), downstream fouling was observed alongside fouling around the pores. The various microscopic observations are useful in elucidating four categories of membrane fouling (a) cake filtration (upstream), (b) pore blocking (inside the pores), (c) colloidal aggregation (downstream) and (d) colloidal streamer (downstream).

Figure 2(a) depicts the typical low pressure cake filtration (upstream) scenario at 120 min of filtration, wherein the foulant materials accumulate on the upstream end of the membrane^14,16,29,33^. Although, cake filtration usually occurs due to the deposition of particles larger than the pore-scale, Fig. 2(a) schematic shows that if the foulants are smaller than the pore-scale, then they can aggregate and be packed to form a barrier at the upstream end of the membrane.

Pore blocking is typically more significant under moderate pressures^17,18,34^; when the solutes are forced through the membranes and adsorb onto the membrane pore walls. Pore-blockage (inside the pores) is seen when PAM only solution is flown through the MMM device. The PAM molecules have a radius of gyration, *R*_*g*_ = 191.9 nm^35^, and their entanglement can completely block the pores during the course of filtration (Fig. 2(b)). Fouling due to PAM was imaged under the same conditions, and although the PAM molecule is not red fluorescent, bright and dark areas can be seen under optical microscopy (Fig. 2(b)). This might be due to higher light scattering from areas which have higher PAM aggregation. Figure 2(b) shows that PAM polymer plugged the pores after 120 mins of operation at 345 mbar.

Higher pressure can further force the foulant materials to flow through the membrane pores and reach at the downstream side of the pores. When PS particles reach at the downstream zone of the pillars, they start aggregating along the flow direction as shown in Fig. 2(c). The colloidal aggregation phenomenon at downstream location^25,31^ is observed to occur concurrently with cake filtration and pore blocking (Fig. 2(c)). Figure 2(c) shows such a fouling process which is accompanied by aggregation of colloidal particles (See Supplementary Video V1) just downstream of the pores which is discussed later.

The colloidal streamer mode of fouling is relatively newly discovered mode of membrane fouling. It was demonstrated recently that in the context of low Reynolds number flows, organic materials such as bacteria can lead to the formation of filamentous structures called ‘streamers’^36^. These filamentous structures can proliferate rapidly in microfluidic devices leading to pervasive colonization and clogging^37,38^ leading up to a catastrophic failure of the device^38,39^. Due to its very nature these streamers can thrive into various sections of a microfluidic device including the downstream sections of filtration systems^36,37,40^. Debnath *et al*.^35^ recently demonstrated that particle laden polymeric flows can also lead to morphologically similar structures as bacterial streamers leading to the generalization of the phenomenon to other colloidal systems. When a mixture like particle laden polymer (PAM(0.2%):PS(0.2%) = 1:4) is filtered through our MMM system, a filamentous compliant structure was formed at downstream of the pillars. This structure is called the ‘colloidal streamer’^41^. Figure 2(d) shows colloidal streamer fouling, which is the second kind of downstream fouling captured at 120 min of filtration at 689 mbar pressure. The formation of the colloidal streamer at downstream location (See Supplementary Video V2) is discussed in details later.

### Constant-pressure filtration

**Figure 3:**
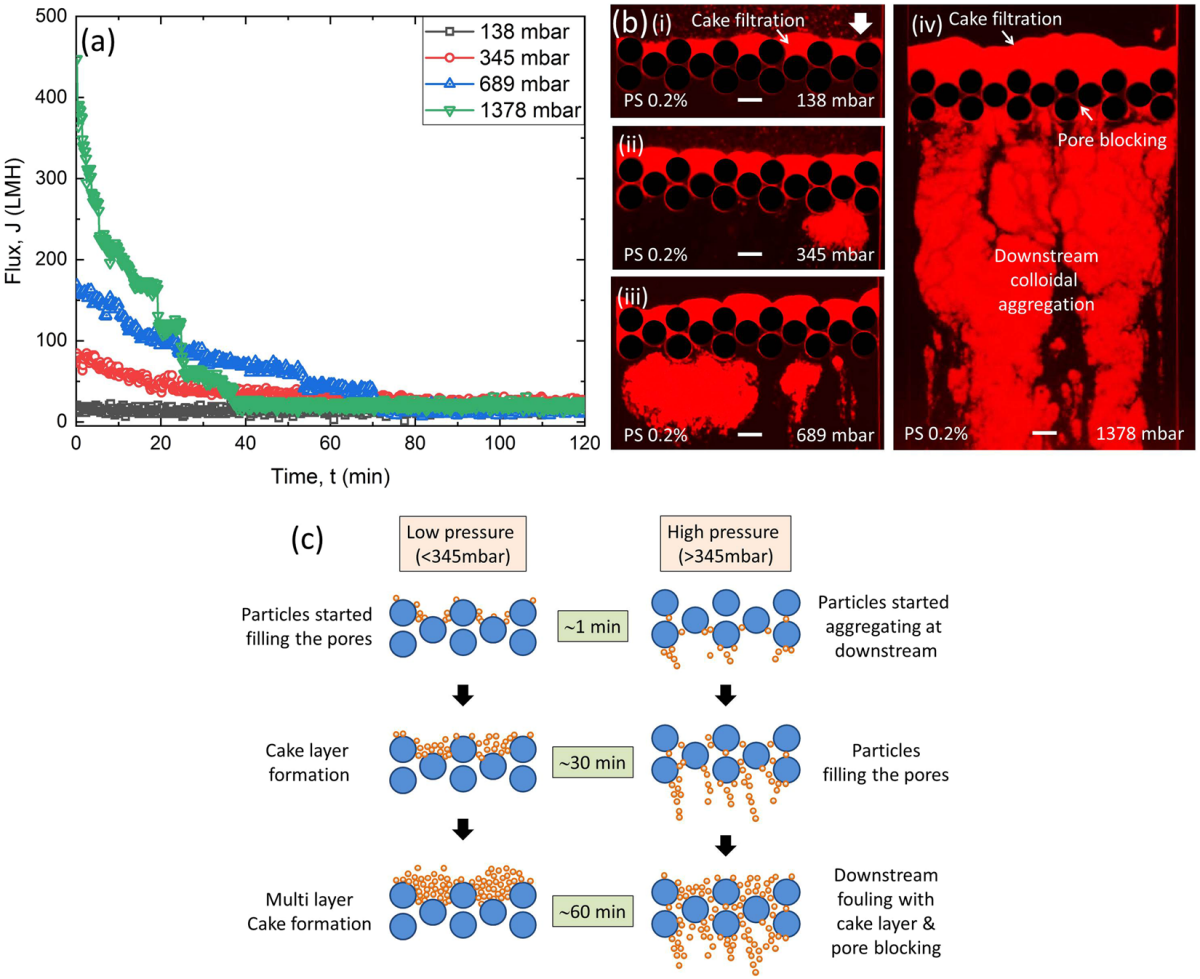
(**a**) Filtration experiments at constant pressure shows decreasing flux for PS 0.2% as fouling progresses with filtration time. **(b)** Corresponding microfluidic images show end-result of fouling (120 min). At low pressures, fouling is more like cake filtration at upstream (i)–(ii). At higher pressures more colloidal aggregation are observed at downstream with simultaneous pore blocking and cake filtration (iii)–(iv). **(c)** Schematic of fouling at low pressure (<345 mbar) and high pressure (>345 mbar) showing the fouling evolution in 60 min.

The effect of constant applied pressure difference on water flux through MMM was studied. The pressure difference across the microfluidic channel was varied from 138 to 1378 mbar. To investigate the effect of colloidal fouling, first only PS 0.2% bead solution was used as feed, and the results are shown in Fig. 3(a). The flux, *J*, is calculated from the direct measurement of the flow rate obtained by MFCS system using the relationship *J* = *Q*/*(w* × *h*), where *Q* is the flow rate (m^3^/s), *w* is the width of the channel (504 µm), and *h* is the height of the pillars (5 µm). Figure 3(a) shows that at 138 mbar the flux is almost constant. Hence, 138 mbar can be considered as a limiting pressure/critical pressure below which fouling does not occur for PS 0.2% solution. In membrane filtration, the critical flux is defined as the permeate flux above which the irreversible membrane fouling occurs^42,43^. The approximate critical flux for our MMM system is, *J*_*cr*_ ∼ 24.62 LMH, calculated as, *J*_*cr*_ = *Q*_*cr*_/*(w* × *h*), where *Q*_*cr*_ = 1.72 × 10^−14^ m^3^/s.

In general, larger particles than the pore size of membrane are blocked on the membrane surface during filtration and form a cake layer^44^. However, smaller particles than membrane pore size can also form a cake layer, when inception of fouling occurs inside the pores and grows to the filter cake on the membrane surface as the membrane pores become narrower over time^44^. From our direct microfluidic observations, low pressures (≤345 mbar) caused pore blocking at the surface of the pores first and developed to a filter cake (Fig. 3(b)(i),(ii)) with time. Figure 3(b) captures filtration fouling with PS 0.2% for filtering over 120 min. This kind of fouling is also in good agreement with *ex-situ* MF/UF filtration results in the literature^15,18^. In contrast, higher pressures (≥689 mbar) led to higher initial flux (∼160 LMH at 689 mbar), but a sharp decline in flux over time, as shown in Fig. 3(a). At higher pressures, the larger hydrodynamic drag force caused the particles to pass through the pores of pillars and aggregate at the downstream zone of pillars (Fig. 3b(iii),(iv)). This type of fouling may occur in the finger-like macrovoids of porous membranes, underneath the top skin layer, when filtered particles diameter is less than the pores of skin layer. It was observed from the online monitoring of the fouling at 1378 mbar (Video V1 and Fig. S3 in Supporting Information) that the fouling started with the colloidal aggregation at downstream zone. It continued for 40 min simultaneously with partial pore blocking and cake layer formation (Fig. S3 in Supporting Information). After that, downstream colloidal aggregation reached a steady state with an increase in the cake layer thickness (Fig. S3 in Supporting Information) for the rest of the filtration process. Fig. 3(c) shows the schematic of the fouling process at low pressure (<345 mbar) and high pressure (>345 mbar) with time. It can be seen from the schematic that at low pressure pore blocking causes the cake layer formation while for higher pressure downstream fouling causes the pore blocking and pore blocking leading to cake layer formation eventually. The constant pressure filtration results were repeated in triplicate (see Fig. S4 in Supporting Information).

At low pressures, we hypothesise that the particles mostly attach to the upstream surface of the pillars as the hydrodynamic drag force could not overcome the attractive surface interaction between particle-PDMS wall (Fig. 3b(i),(ii)). The zeta potential measurement showed a strong attractive electrostatic force exits between positively charged PS particles (*ξ*_*PS*_ ∼ +30 mV at pH 7) and negatively charged PDMS surface (*ξ*_*PDMS*_ ∼ −45 mV at pH 7). As a result, cake filtration is generally exhibited at low pressures (≤345 mbar). However, at higher pressures (≥689 mbar), hydrodynamic drag force might overcome the interaction energy between particles and particle-PDMS surface. Higher shear stresses and advection rates lead to particle aggregation downstream of the pillars (Fig. 3(b)(iii)). At 1378 mbar pressure, the inception of fouling occurred at downstream location (Supplementary Video V1 and Supplementary Fig. S3). The downstream colloidal aggregation continued with partial filling of the pore space. When the particles started filling the pores, flow distribution was no longer uniform across the membrane width due to the constricted pore space. Hence, the local velocity of water increased for partially open pores to maintain a constant pressure difference. The increase in the local velocity caused primary and secondary water channel formation by continuous aggregation/sloughing of particles more towards the flow directions^45^ (Supplementary Video V1). Hence, as filtration proceeded, the detachment of the particles occurred by sloughing and the higher pressure difference eventually resulted in the steady colloidal aggregation at downstream with pore blocking and cake layer after 120 min of filtration (Fig. 3b(iii),(iv), Supplementary Video V1).

We have also conducted constant pressure (689 mbar) experiments to examine the effect of changing PS concentrations (PS 0.02%, 0.4% and PS 1%) on fouling propensity. As can be observed in Fig. S5, all of these experiments had led to downstream fouling. Our experiments indicate that the qualitative nature of fouling does not change due to change in concentrations but the same fouling behaviour seems to occur faster at the higher concentrations.

**Figure 4:**
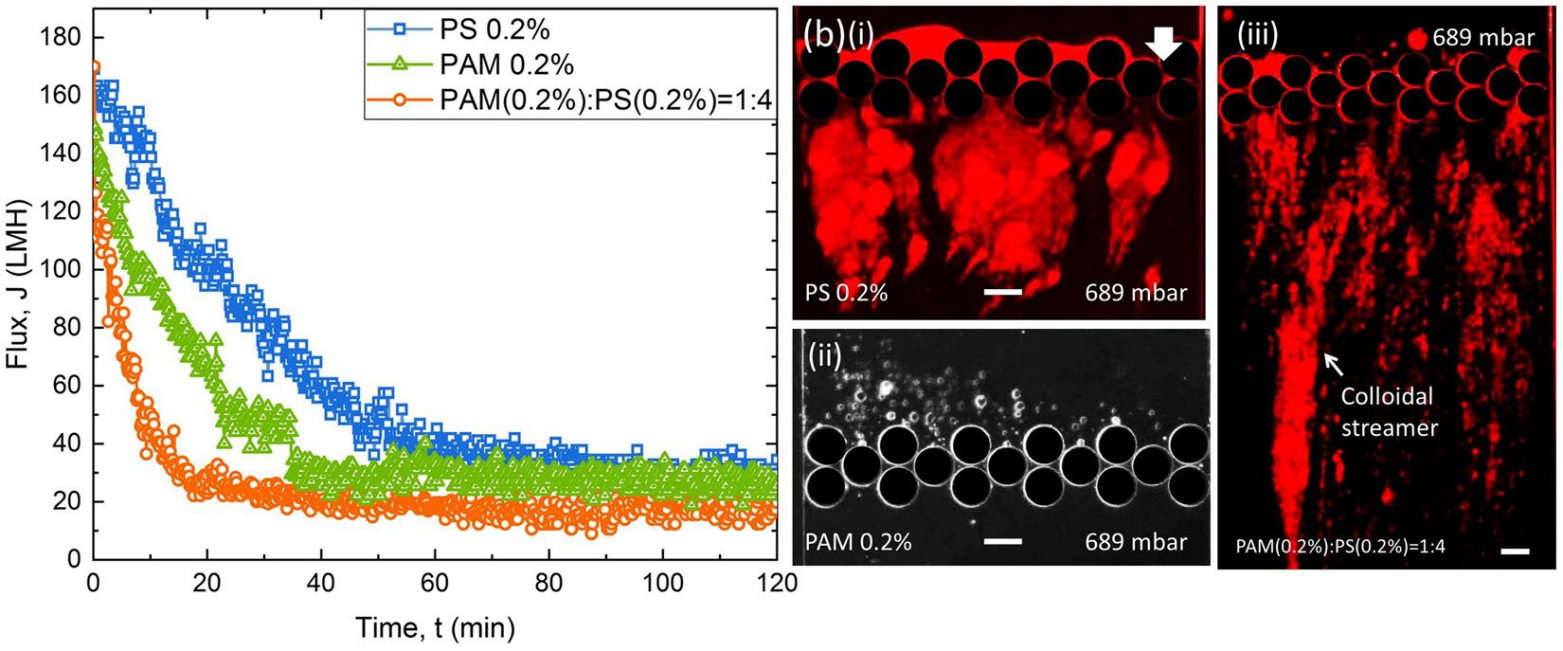
Comparison of the constant-pressure fouling behaviour for three types of foulants at the same pressure (689 mbar). **(a)** Flux vs. time plot showing more severe flux decline for the case of combined fouling (PAM (0.2%):PS (0.2%) = 1:4) than individual organic and colloidal fouling (PAM 0.2% & PS 0.2%), **(b)** corresponding microfluidic images at the end of the filtration process showing (i) Colloidal aggregation at downstream with partial pore blocking and cake filtration for PS filtration, (ii) pore blocking for PAM filtration, and (iii) streamer formation at downstream for the case of combined fouling.

To further investigate the effects of physiochemical interactions on fouling, we have extended our experiments to three different types of foulants at the same constant pressure. The constant pressure experiments were conducted using particle solution (PS 0.2%), polymer solution (PAM 0.2%) and a mixture of PAM(0.2%):PS(0.2%) = 1:4 (v/v) to investigate colloidal, organic and combined fouling, respectively. Microfluidic filtration at low pressures did not show significant difference in permeate flux for all types of fouling, whereas, at a pressure of 689 mbar, the decline was found to be more severe for the case of combined fouling than the individual organic and colloidal fouling (Fig. 4(a)). Starting with same initial flux (∼160 LMH), the approximate filtration time for the permeate flux to become steady were 20, 40, and 60 min for the combined fouling, organic fouling, and colloidal fouling, respectively (Fig. 4(a)). Such a severe flux decline in the case of combined fouling was also observed in *ex-situ* membrane processes^46,47^. It was found that PS 0.2% resulted in colloidal aggregation at downstream with partial pore blocking and cake filtration (Fig. 4b(i)) and PAM 0.2% mostly caused pore blocking (Fig. 4(b)(ii)). When a combination of PAM and PS were injected as feed (PAM(0.2%):PS(0.2%) = 1:4), slender filamentous structures were formed around the pillars and at downstream of the pillars (Fig. 4(b)(iii)). Debnath *et al*.^11^ named this structure as ‘colloidal streamer’ and this occurs due to synergistic effect resulting from a bridging of PS beads by the polymeric molecules and subsequent adhesion and shearing due to hydrodynamic forces^35^. The attraction between positive PS (*ξ*_*PS*_∼ +30 mV at pH 7) and negative PAM molecules (*ξ*_*PAM*_ ∼ −30 mV at pH 7), and the negative PDMS surface (*ξ*_*PDMS*_ ∼ −45 mV at pH 7) caused PAM + PS floc formation and attachment to the surface leading to the streamer fouling at downstream. The dynamics of colloidal streamers is discussed elsewhere^11,45^ and here we focus only on the fouling characteristics of streamers. At 689 mbar pressure, colloidal streamer formation was observed instantaneously (see Supplementary Video V2). With time, the streamer accumulated more mass, became thicker and extended up to ∼500 µm from the pillar surface towards the flow direction (see Supplementary Video V2). Interestingly, within 20 min the flux reached the steady state but the streamer fouling continued (Fig. 4(a) and Supplementary Video V2). It was also observed that the process of downstream streamer formation and subsequent streamer breaking were observed continuously during the filtration period^37^. Here, PAM molecules were invisible under red fluorescence microscopy. The constant pressure filtration results were repeated in triplicate (see Figs S4 and S6 in Supporting Information). When the repeatability data were fitted to the exponential decay equation (Supplementary Figs S4, S6 and Table S1), the coefficient of determination (*R*^2^) values showed better agreement for the case of organic fouling (PAM 0.2%) and combined fouling (PAM 0.2% + PS 0.2% = 1:4) than the colloidal fouling (PS 0.2%) for the same pressure (689 mbar) experiments. The analytical modelling of the downstream and streamer types of fouling is the topic of our ongoing research.

Besides, the primary and secondary water channel formation was more prominent at the end (120 min) of filtration (Supplementary Video V2 and Supplementary Fig. S7).

Different membrane fouling mechanisms are discussed in the literature^22,23^. Ho *et al*. discussed the combined cake filtration and pore blocking model for protein fouling in a MF system^48^. But the cake filtration and pore blocking model cannot explain the flow/detachment behaviour of particles during fouling process. These can further be explained by comparing different fouling scenarios and examining the fouling percentage which are discussed next.

**Figure 5:**
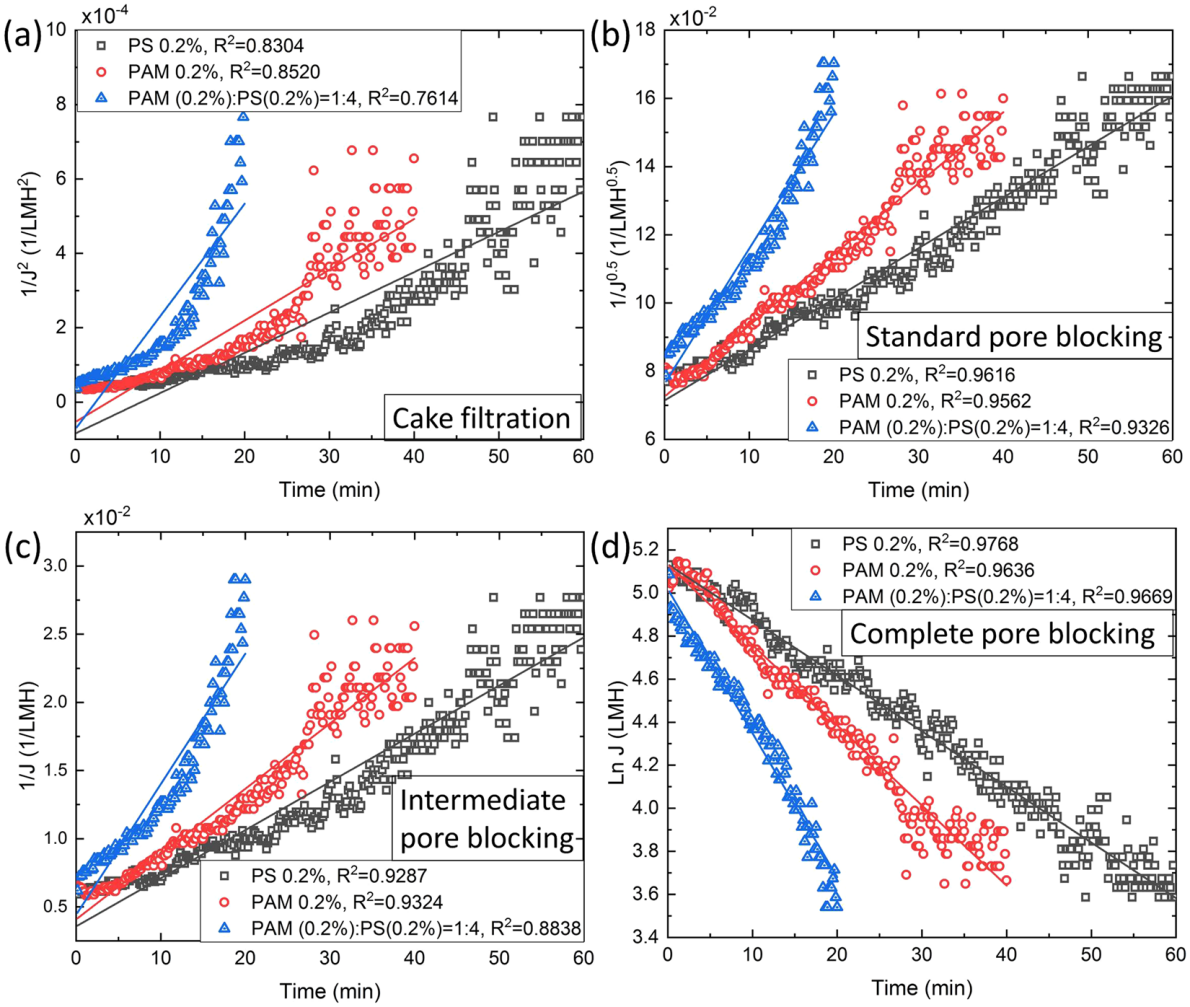
Flux vs time plot for the three foulants using Hermia model at constant pressure (689 mbar). Curves are fitted using regression analysis where *R*^2^ value represents regression coefficient. The flux trends are compared to Hermia’s model for **(a)** cake filtration, **(b)** standard pore blocking, **(c)** intermediate pore blocking, **(d)** complete pore blocking. From the regression analysis, the maximum value of *R*^2^ indicates that the fouling is complete pore blocking for the three foulants.

In order to reveal more information about the governing fouling mechanisms exhibited by different foulants, the experimental constant pressure flux results (689 mbar) were fitted to the linear equations derived for constant pressure filtration by Hermia model^22^. Hermia categorised four kinds of fouling: cake filtration, standard pore blocking, intermediate pore blocking and complete pore blocking with the decline in flux^22^. Figure 5 shows that our results were more in agreement with the complete pore blocking (Fig. 5(d)) for the filtration results with the three foulants at 689 mbar pressure. The microfluidic observations are in consonance with the findings. For PS particles partial cake filtration and pore blocking were observed and hence Fig. 5(a–c) show a less degree of match as compared to Fig. 5(d). However, for the combined foulant of PAM and PS, we know from our observations that streamer formation occurred downstream of the pillar wall. Interestingly, the fouling due to streamers is also consistent with the time signature of complete pore blocking. This suggests that streamer formation occurs in such a manner that complete pore blocking is achieved simultaneously. However, from the pore-scale perspective, streamer led-clogging is a different kind of fouling as compared to complete pore blocking. This suggests that this is an entirely different fouling mechanism and needs to be studied in more detail. While, some early work in the area of modelling of streamer-clogging has taken place^39,49^, more extensive modelling work is desirable.

**Figure 6:**
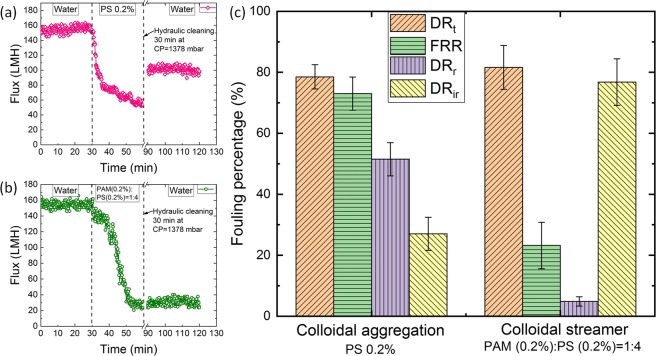
Fouling percentage evaluation for colloidal aggregation and streamer fouling at the same pressure 689 mbar. At first, the dead-end filtration is performed for the clean system with pure water for 30 min, next the fouling experiments are performed for another 30 min. After that, a hydraulic cleaning is performed to clean the chip for another 30 min at higher pressure. Then, pure water filtration is performed again for 30 min. Figure **(a)** and **(b)** show the corresponding flux for PS 0.2% and PAM(0.2%):PS(0.2%) = 1:4, respectively. **(c)** Represents the corresponding fouling percentage contributions for the same. Reversible flux decline ratio (*DR*_*r*_) is decreased by ∼24% than irreversible flux decline ratio (*DR*_*ir*_) for colloidal aggregation and irreversible flux decline ratio (*DR*_*ir*_) is increased by ∼72% than reversible flux decline ratio (*DR*_*r*_) for streamer fouling.

To analyze the percentage contributions of the colloidal aggregation and colloidal streamer fouling, flux recovery tests were performed using PS 0.2% and the combined solution (PAM (0.2%):PS (0.2%) = 1:4) (Fig. 6(a,b)). First, pure water flux was (*J*_*w1*_) obtained at 689 mbar pressure for 30 min. Next, fouling experiments were conducted at the same pressure for another 30 min (*J*_*wf*_). A hydraulic washing was then performed for another 30 min to clean the system at a higher pressure (1378 mbar) than filtration pressure (689 mbar). Finally, pure water flux (*J*_*w2*_) was obtained again at 689 mbar (Fig. 6(a,b)). The experimental results show more reversible fouling for colloidal aggregation; more irreversible fouling for colloidal streamer fouling (Fig. 6(c)). Here, several evaluating parameters are defined as reversible flux decline ratio (*DR*_*r*_), irreversible flux decline ratio (*DR*_*ir*_), flux recovery ratio (*FRR*) and total flux decline ratio (*DR*_*t*_). We have calculated *DR*_*r*_, *DR*_*ir*_, *FRR* and *DR*_*t*_ with the formulas: (*J*_*w2*_ − *J*_*wf*_)/*J*_*w1*_, 1 − *J*_*w2*_/*J*_*w1*_, *J*_*w2*_/*J*_*w1*_, 1 − *J*_*wf*_/*J*_*w1*_, respectively following the similar process in membrane filtration^50,51^. From Fig. 6(c), the reversible flux decline for the colloidal aggregation (PS 0.2%) was recovered more (*FRR* = 72.98%) and the reversible deposition of PS 0.2% probably released from the membrane surface by hydraulic washing. The reversible fouling for PS 0.2% accounted for *DR*_*r*_ = 51 % from *DR*_*t*_ = 78% overall fouling. However, streamer fouling was found to be mainly irreversible (*DR*_*ir*_ = 76% from *DR*_*t*_ = 81% overall fouling) and less recovery of the flux obtained (*FRR* = 23.21%) due to the direct attachment and blockage of the pores, which were difficult to recover.

The result can be explained by the fragility of the flocs^31^. The PS particles are spherical (diameter, 0.2 μm) in shape. The PS aggregates are smaller, loosely packed and fragile compared to the PAM + PS flocs which are bigger, compact, cohesive, and highly deformable^31,52^. As a result, the higher background shear force led to the sloughing of the loosely packed PS aggregates through the pillar pores. However, some particles/aggregates remained attached to the pillar walls^53^. The remaining few attached particles contributed to the very less irreversible fouling for PS 0.2% (Fig. 6(c)). In case of PAM + PS flocs, due to the compact, cohesive and deformable nature of the flocs, higher background shear force could not break or wash away the pores completely, contributing mostly to the irreversible fouling (Fig. 6(c)).

## Conclusion

The dynamics of the fouling formation in a MMM device were presented by varying the hydrodynamic conditions and solution chemistry. Overall colloidal fouling scenarios were divided into four major categories: cake filtration, pore blocking, colloidal aggregation and colloidal streamer. Different fouling scenarios were captured by microfluidic observations at pore scale in real-time analysis. Constant pressure experiments showed more decline in flux due to colloidal aggregation and colloidal streamer fouling at a higher pressure. Similar to the result obtained by commercial membrane fouling, the combined fouling like colloidal streamer caused more fouling in the MMM system than individual fouling. Colloidal streamer fouling is considered as a special kind of fouling which contributes more to irreversible fouling and it does not follow the Hermia model. In summary, our experimental technique models a dead-end membrane module for the microscopic fouling study and illustrates the importance of collective interplay of hydrodynamics and physiochemical interactions to establish different fouling scenarios at the pore scale. In conclusion, significant fouling may also occur due to the attachment of particles at the downstream end of membrane pores which cannot be ignored.

## Materials and Methods

### Experimental

The schematic of the experimental setup is shown in Fig. 1(a). For the microfluidic dead-end filtration, a pressure-driven flow was created in the microchannel by using microfluidic flow control system (MFCS) (Fluigent, MA, USA). The feed was connected to the inlet via a flow unit (Fluigent, MA, USA) and permeate was collected from the outlet of the MMM system (Fig. 1(a)). The MMM filtration experiments were performed at a constant-pressure difference (Δ*P*), maintained by the microfluidic pressure controller (MFCS-EZ) (Fluigent, MA, USA). The corresponding volumetric flow rate (*Q*) was measured directly from the flow-rate-control-module software (Fluigent, MA, USA). All experiments were conducted at creeping flow condition (*Re* < 1) with maximum fluid velocity *v*_*max*_ ~ 6.84 × 10^−4^ m/s, considering the channel hydraulic diameter *d*_*h*_ ~ 2.86 μm. Three different foulants (polymer, particles and a mixture of polymer and particles) were tested at neutral pH condition. All experiments were performed at room temperature and repeated thrice. The MFCS (Flow unit) was never let to dry & cleaned thrice with ethanol solution before changing any feed sample.

### Microfabrication

The microfluidic device was fabricated by conventional photolithography technique using polydimethylsiloxane (PDMS, Sylgard 184, Dow Corning, NY, USA) with the membrane mimic design. The required membrane design was replicated from a 4″ silicon master mold. The microfluidic design consists of a straight channel with a set of staggered array of pillars near the mid-section (Fig. 1(b)), which acts as a MF membrane mimic. The staggered arrangement of pillars has a height *h* = 5 µm and diameter *d* = 50 µm, and the gap between pillars *p* = 2 µm (Fig. 1(b)). Hence, the device provides a pore size which is comparable to an MF membrane pore (0.1–10 μm). The thickness, *t*, of the membrane is 102 µm and the width, *w*, of the microchannel is 504 µm, as shown in Fig. 1(b). The inlet and outlet pores were drilled carefully and the PDMS stamps and coverslip were bonded together by using oxygen plasma-activated bonding at 500mTor pressure for 30 seconds. Next, they were annealed at 80 ºC for 1 hour to ensure proper bonding. Additional details about the fabrication process is provided elsewhere^38^. The zeta potential of the PDMS surface (*ξ*_*PDMS*_) was measured to be ∼−45 mV at pH 7 after plasma treatment (SurPASS^TM^ 3, Anton Paar, Graz, Austria).

### Microscopy

The membrane mimic microfluidic device was placed on a stage of an inverted optical (Nikon Eclipse Ti) microscope and fluorescent imaging was performed by using a Texas Red filter cube (Nikon) (Fig. 1(a)). Fluorescence microscopy technique enabled processing the real-time imaging and videography by using the image-processing module in the Nikon NIS-Element AR software interface. Scanning electron microscopy (SEM) images were taken using a field emission scanning electron microscope (Zeiss, Oberkochen, Germany). Each sample was carbon coated (Denton Vacuum, Desk II, Moorestown, New Jersey) before SEM imaging. Images were taken at 20 kV with an in-lens secondary electron detector (Fig. 1(b)).

### Foulant materials

Three types of synthetic wastewater solutions were prepared. (1) Polymer solution (PAM 0.2% w/w): polymer solution was prepared by dissolving 1 g of anionic polyacrylamide (PAM: A-8354, 22 MDa, Kemira, AB, Canada) into 500 mL of DI water. Then the solution was stirred at 600 rpm for more than 3 hr using a magnet stirrer (Caframo, ON, Canada) to ensure homogeneous mixing. The zeta potential of PAM solution (*ξ*_*PAM*_*)* was measured to be ∼−30 mV at pH 7 (Malvern Zetasizer). (2) Particle solution (PS 0.2% w/w) was prepared by dissolving 200 nm amine-modified polystyrene beads (excitation at 580 nm, emission at 605 nm, Life Technologies, ON, Canada) into Millipore water. Zeta potential measurement showed that PS solution (*ξ*_*PS*_*)* was ∼+30 mV at pH 7 (Malvern Zetasizer). Red fluorescent PS particles appeared red under fluorescence microscopy. (3) A mixture of polymer and particle solution (PAM + PS (0.2%) = 1:4 v/v). Based on our previous study^35^, the optimum ratio of PAM (0.2% w/w) and PS (0.2% w/w) solutions to observe colloidal streamer formation was found to be 1:4 (v/v). This ratio was selected in the present work to investigate the combined colloidal and organic fouling on fouling propensity in the MMM device.

## Supplementary information

Investigating fouling at the pore-scale using a microfluidic membrane mimic (MMM) filtration system

Dynamics of fouling by filtering PS 0.2% at 1378 mbar pressure in the MMM device

Dynamics of fouling by filtering PAM (0.2%):PS (0.2%)=1:4 at 689 mbar pressure in the MMM device
