# Supplementary material for: Investigating fouling at the pore-scale using a microfluidic membrane mimic filtration system
Source: Sci Rep. 2019 Jul 22;9:10587. doi: 10.1038/s41598-019-47096-6 (PMC6646390; doi:10.1038/s41598-019-47096-6)
Supplement: Supplementary file 1 — Investigating fouling at the pore-scale using a microfluidic membrane mimic (MMM) filtration system [file 41598_2019_47096_MOESM1_ESM.docx]

**Supplementary information for**

**Investigating fouling at the pore-scale using a microfluidic membrane mimic (MMM) filtration system**

**Nandini Debnath^a^, Aloke Kumar^b^, Thomas Thundat^c,d^, Mohtada Sadrzadeh^a,^***

^a^Department of Mechanical Engineering, 10-367 Donadeo Innovation Centre for Engineering, Advanced Water Research Lab (AWRL), University of Alberta, Edmonton, AB T6G 1H9, Canada.

^b^Department of Mechanical Engineering, Indian Institute of Science, Bangalore, India

^c^Department of Chemical and Materials Engineering, University of Alberta, Edmonton T6G 2G8, Canada

^d^Department of Chemical and Biological Engineering, School of Engineering and Applied Sciences, University of Buffalo, Buffalo NY, 14260

*Corresponding author email: [sadrzade@ualberta.ca](mailto:sadrzade@ualberta.ca).

1. **Pure water flux vs pressure plot**


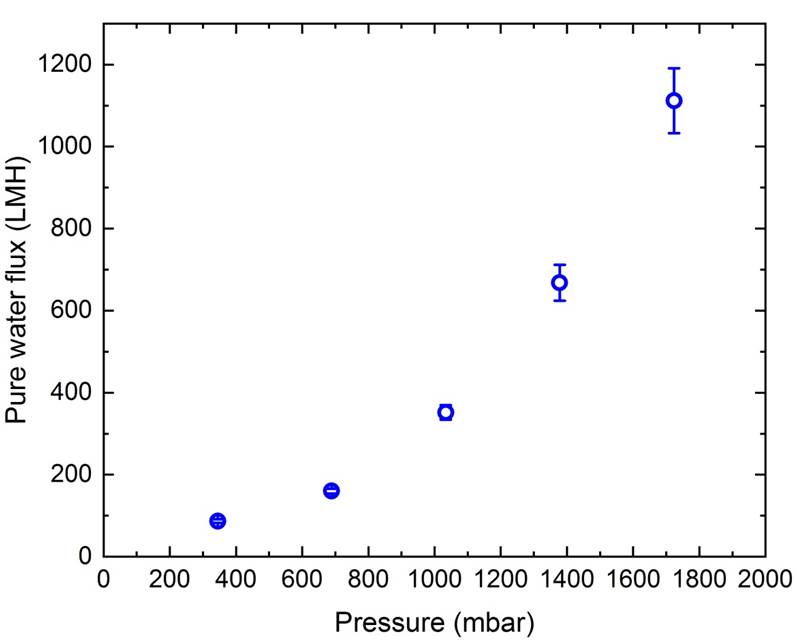


**Figure S1**: Pure water flux as a function of pressure.

1. **Repeatability experiments for water**

**
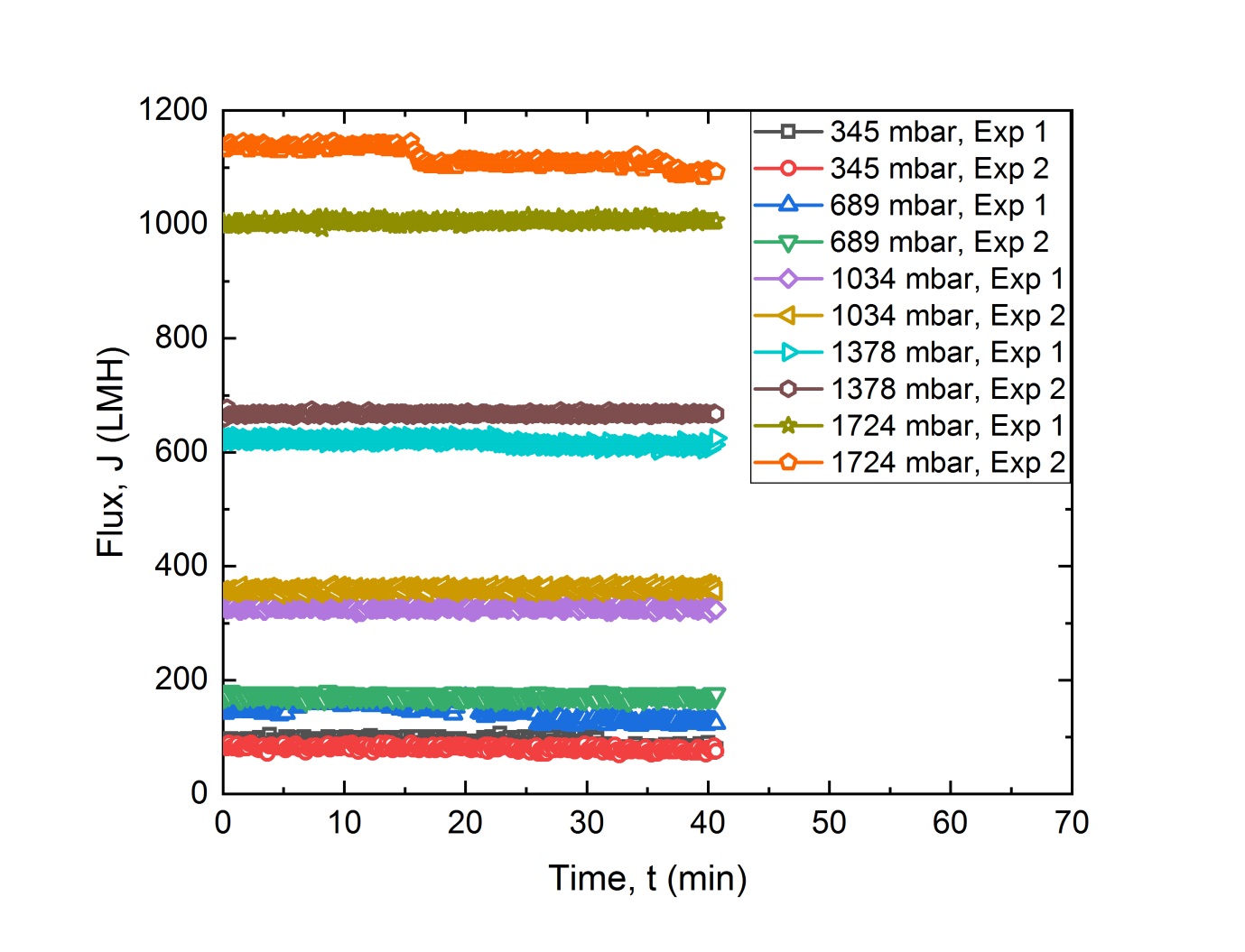
**

**Figure S2.** Constant pressure repeatability experiments for water at different pressure.

1. **Constant pressure experiments for PS 0.2%**

**
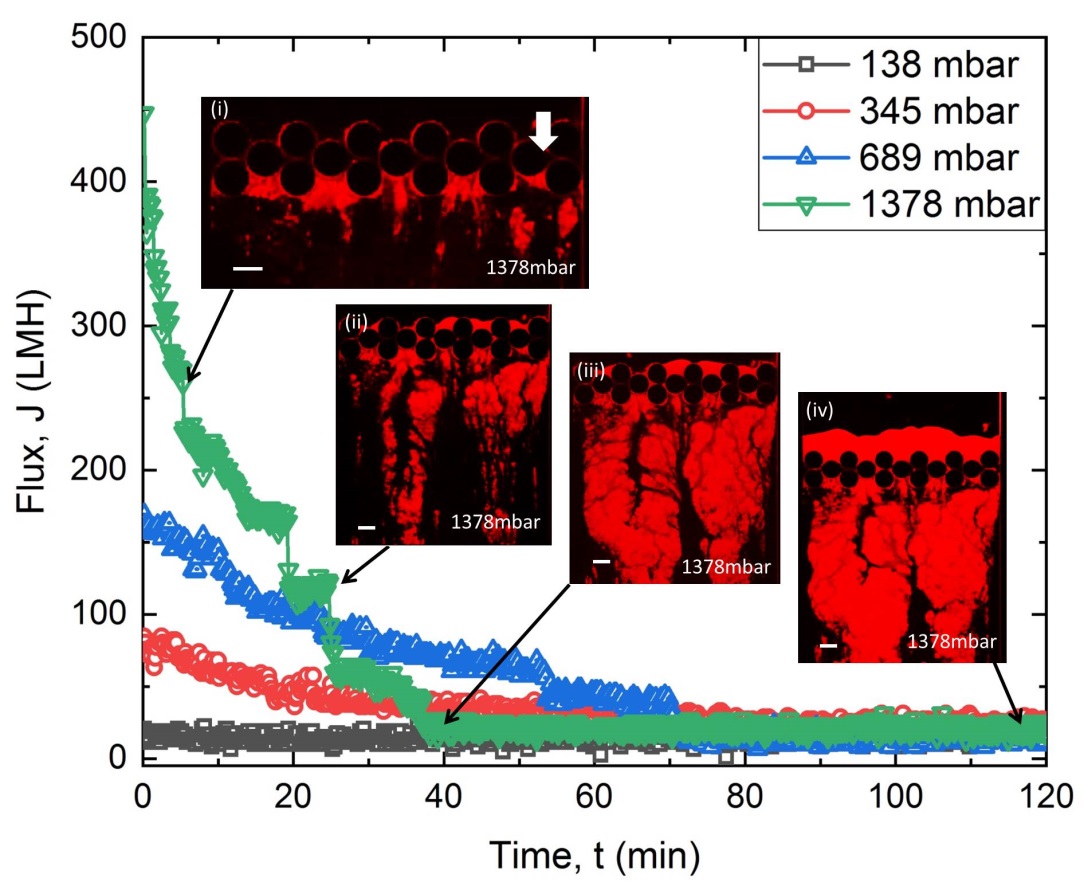
**

**Figure S3.** Constant pressure experiments at 1378 mbar pressure for the PS 0.2% solution show sharp decline in flux compared to lower pressure. **(i)** Microfluidic fouling started with colloidal aggregation at downstream. **(ii)-(iii)** Downstream colloidal aggregation fouling continued with partial pore fouling and filter cake till 40 min of filtration. **(iv)** A significant increase in cake layer thickness is observed till 120min of filtration.

1. **Constant pressure repeatability experiments (PS)**

**
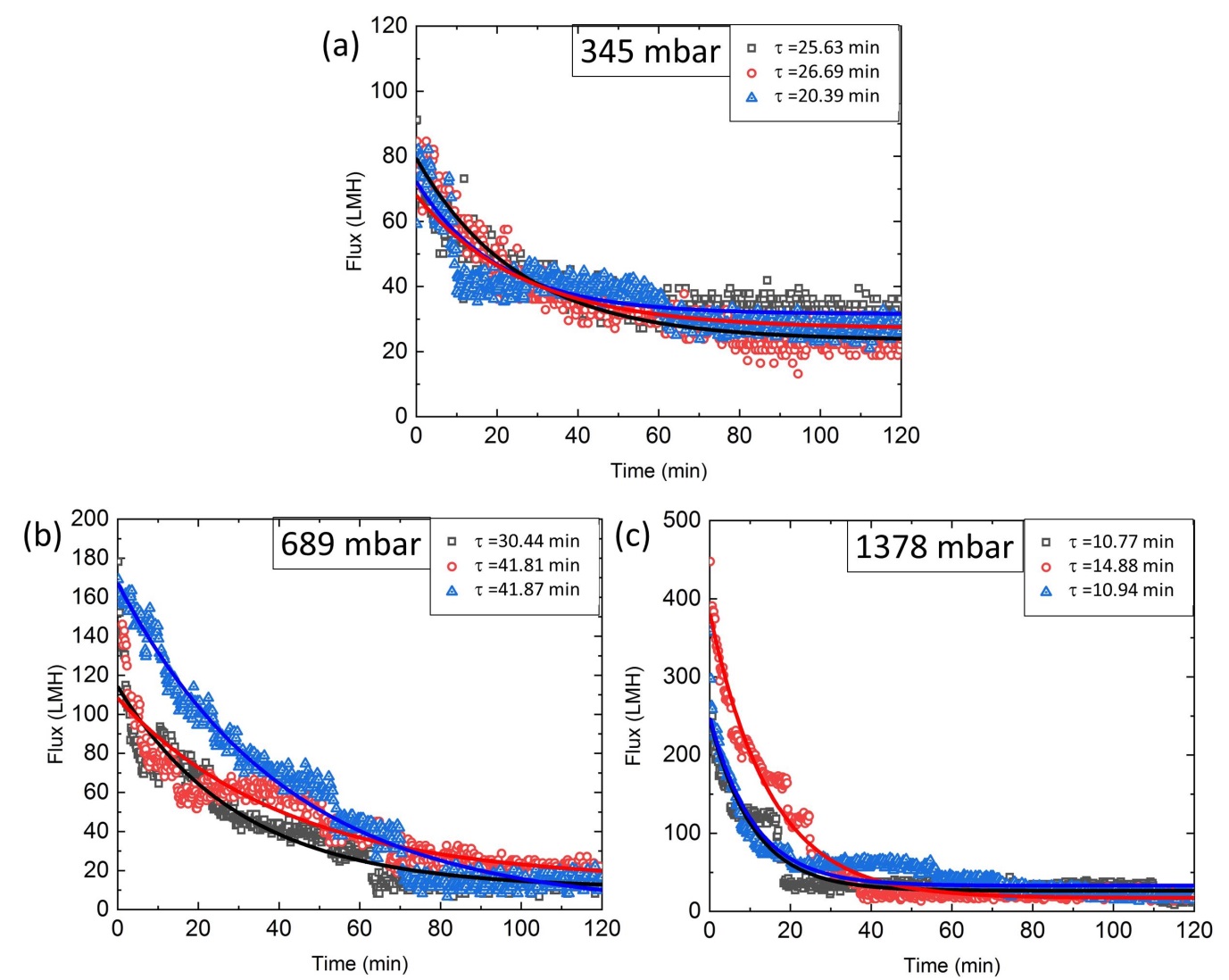
**

**Figure S4.** Repeatability experiments at different constant pressure with PS 0.2% solution. The repeatability curves are fitted to the same exponential decay equation shown in table S1.

1. **Effect of changing concentration for PS**


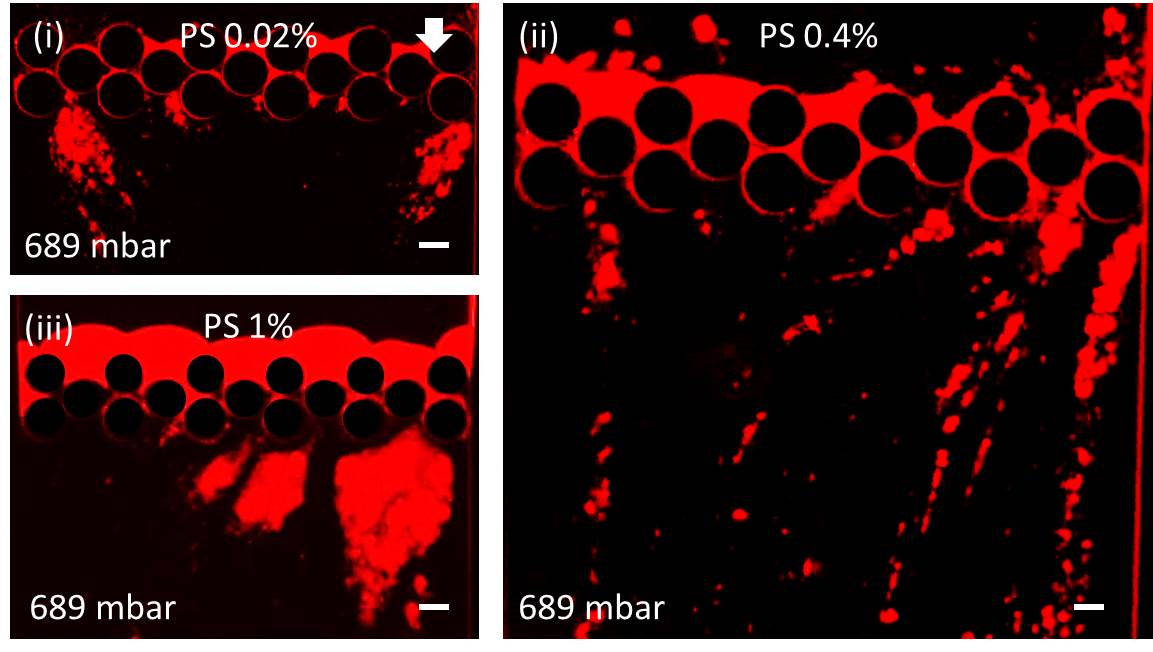


**Figure S5.** Constant pressure fouling scenarios of PS at 689mbar pressure for different concentrations **(i)** PS 0.02% **(ii)** PS 0.4% and **(iii)** PS 1% after 60 min of filtration.

1. **Constant pressure repeatability experiments (PAM, PAM+PS)**


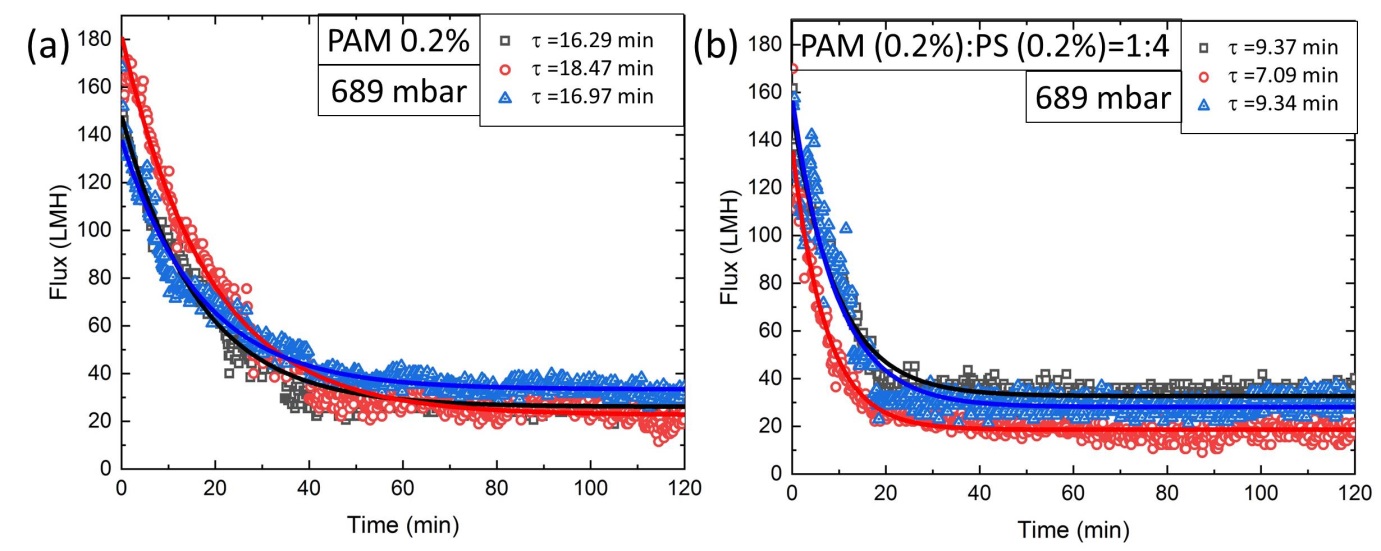


**Figure S6.** Repeatability experiments at constant pressure (689 mbar) for (a) PAM 0.2% solution and (b) downstream colloidal streamer formation with PAM (0.2%):PS (0.2%)=1:4, combined solution filtration.

1. **Table S1.** Repeatability data fitted to the exponential decay equation and the corresponding values of the parameters at variable constant pressure difference. The table corresponds to fits shown in Fig. S4 and S6.

| $\boldsymbol{J=}\boldsymbol{A}_{\boldsymbol{0}}\boldsymbol{+A}\boldsymbol{e}^{\boldsymbol{-}\left( \boldsymbol{t/} \right)}$ | | | | | | |
| --- | --- | --- | --- | --- | --- | --- |
| ΔP (mbar) | **Foulants** | **Legend** | $\boldsymbol{\tau}$ **(min)** | **A** | **A_0_** | ***R^2^* (Goodness of fit)** |
| 345 | PS 0.2% | 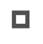 | 25.63 | $5.59\times{10}^{4}$ | $2.34\times{10}^{4}$ | 0.93 |
|  | PS 0.2% | 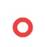 | 26.69 | $4.09\times{10}^{4}$ | $2.70\times{10}^{4}$ | 0.80 |
|  | PS 0.2% | 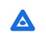 | 20.39 | $4.07\times{10}^{4}$ | $3.14\times{10}^{4}$ | 0.88 |
| 689 | PS 0.2% | 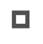 | 30.44 | $1.03\times{10}^{5}$ | $1.07\times{10}^{4}$ | 0.92 |
|  | PS 0.2% | 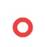 | 41.81 | $9.38\times{10}^{4}$ | $1.45\times{10}^{4}$ | 0.87 |
|  | PS 0.2% | 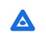 | 41.87 | $1.67\times{10}^{5}$ | $5.15\times{10}^{2}$ | 0.97 |
| 1378 | PS 0.2% | 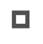 | 10.77 | $2.19\times{10}^{5}$ | $2.63\times{10}^{4}$ | 0.89 |
|  | PS 0.2% | 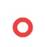 | 14.88 | $3.65\times{10}^{5}$ | $1.71\times{10}^{4}$ | 0.98 |
|  | PS 0.2% | 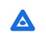 | 10.94 | 2.16$\times{10}^{5}$ | $3.27\times{10}^{4}$ | 0.88 |
| 689 | PAM 0.2% | 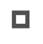 | 16.29 | 1.22$\times{10}^{5}$ | $2.60\times{10}^{4}$ | 0.97 |
|  | PAM 0.2% | 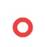 | 18.47 | $1.59\times{10}^{5}$ | $2.26\times{10}^{4}$ | 0.98 |
|  | PAM 0.2% | 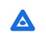 | 16.97 | $1.04\times{10}^{5}$ | $3.33\times{10}^{4}$ | 0.97 |
| 689 | PAM 0.2%+PS 0.2% | 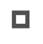 | 9.37 | $1.20\times{10}^{5}$ | $3.26\times{10}^{4}$ | 0.96 |
|  | PAM 0.2%+PS 0.2% | 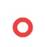 | 7.09 | $1.18\times{10}^{5}$ | $1.85\times{10}^{4}$ | 0.96 |
|  | PAM 0.2%+PS 0.2% | 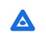 | 9.34 | $1.29\times{10}^{5}$ | $2.80\times{10}^{4}$ | 0.92 |

1. **Water channel formation**

An interesting microfluidic phenomenon, water channel formation was observed for constant pressure filtration. When particles started accumulating inside the inner surface of the pillars, as filtration proceeded, floc propagated from blocking the pore space to a filter cake at low constant pressure (≤345mbar). However, at high pressure (≥689mbar), the flow distribution was no longer homogeneous due to the partial blocking of pores. To maintain the constant volume flow conditions or pressure, there remained some fragile flocs or open channels where the local flow velocity was increased. As a result, the increase in velocity prevented complete pore blocking and caused water channel formation. Water channel formation was observed for the case of bacterial streamer formation earlier^1^. Similar behavior was observed by Lohaus et al. when they performed the numerical simulation with 5µm PS particles in a microchannel^2^ (Figure S7 (a)(i) & (ii)). Interestingly, they found particles accumulating on the center-left channel where there was a prominent open channel with no fouling at the right channel (Figure S7 (a)(i) & (ii))^2^. However, their observation had no experimental evidence. Figure S7 (b) and (c) show water channel formation for colloidal aggregation and streamer formation, respectively. Based on our microfluidic observations, the formation of these water channels were consistent throughout all experiments, especially for the case of downstream fouling at high constant pressures (Figure 3(b)(iii) & (iv), 4(b)(i) & (iii) and video V1, V2). From Figure S7 (b) & (c), primary water channels are in the same direction as flow while secondary water channels are generating from primary. Hence, the formation of primary and secondary water channels not only indicated the particles settling but also they were responsible for the fouling deposition at the downstream side.


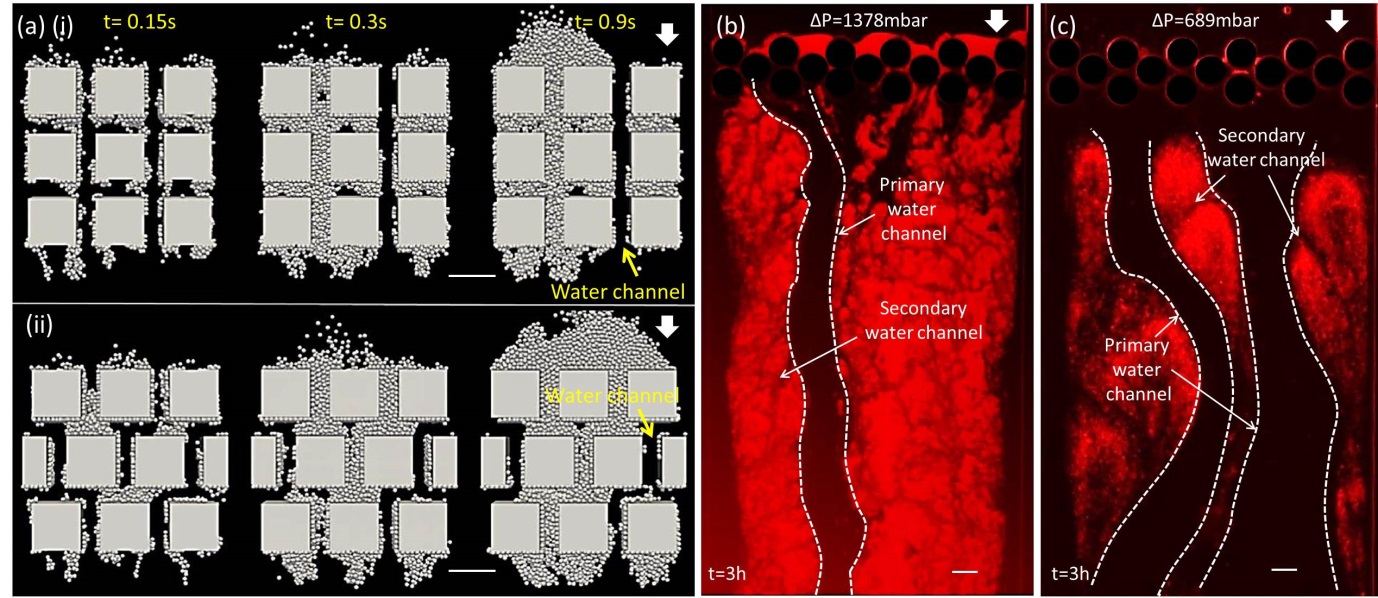


**Figure S7.** **(a)** Water channel formation observed by Lohaus et al. in numerical simulation for the square connected **(i)** and staggered **(ii)** arrangement of pillars^2^. Scale bars are 50µm. (Copyright 2018, reproduced with permission from Journal of Membrane Science). Water channel formation for **(b)** colloidal aggregation (PS 0.2%) at 1378mbar pressure and **(c)** colloidal streamer (PAM (0.2%):PS (0.2%) =1:4) at 689mbar pressure after 180 min of filtration. Primary water channels (marked with white dash line) and secondary water channels are shown with arrows. Scale bars are 50µm.

**Supplementary video V1**

The supplementary video V1 shows the dynamics of fouling by filtering PS 0.2% at 1378 mbar pressure in the MMM device.

**Supplementary video V2**

The supplementary video V2 shows the dynamics of fouling by filtering PAM (0.2%):PS (0.2%)=1:4 at 689 mbar pressure in the MMM device.

**References**

1. Hassanpourfard, M., Ghosh, R., Thundat, T. & Kumar, A. Dynamics of bacterial streamers induced clogging in microfluidic devices. *Lab Chip* **16,** 4091–4096 (2016).

2. Lohaus, J., Perez, Y. M. & Wessling, M. What are the microscopic events of colloidal membrane fouling? *J. Memb. Sci.* **553,** 90–98 (2018).
